# Supplementary material for: Chemical Constituents from Streblus taxoides Wood with Their Antibacterial and Antityrosinase Activities Plus in Silico Study
Source: Antibiotics (Basel). 2023 Feb 3;12(2):319. doi: 10.3390/antibiotics12020319 (PMC9952338; doi:10.3390/antibiotics12020319)
Supplement: Supplementary file 1 [file antibiotics-12-00319-s001.zip › antibiotics-2156140-supplementary.pdf]

Supplementary Materials

Table S1.A list of isolated compounds obtained from *S. taxoides*, downloaded from the PubChem database.

No.	Ligand	PubChem CID	Remark
1	Omega-Hydroxymoracin C	-	self-made from Moracin C
2	Moracin M	185848	-
3	Moracin C	155248	-
4	3,3',4,5'-Tetrahydroxybibenzyl	152444	-
5	piceatannol	667639	-

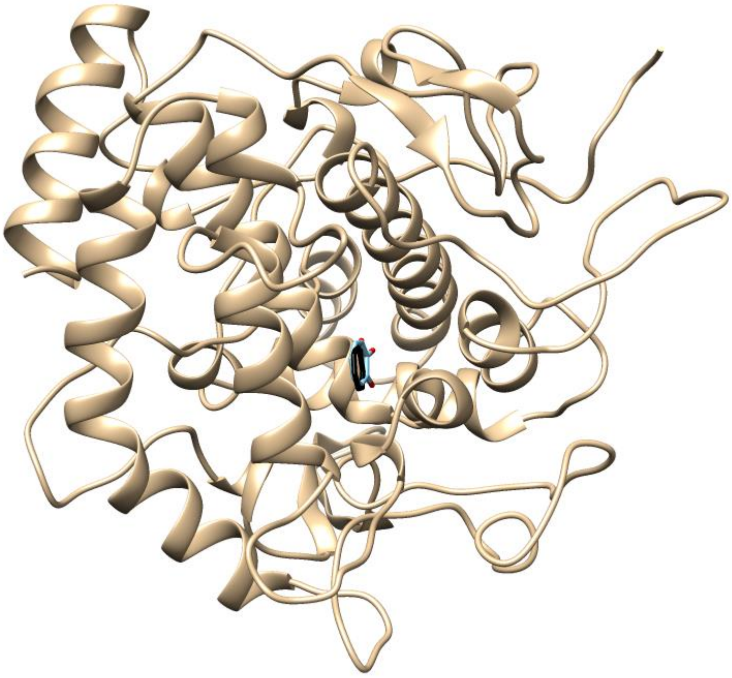

Figure S1. Docking protocol validation. Native tropolone structure (black color) locates in its original position. Re-docked tropolone (blue color) into its original pose overlays over native tropolone structure. The RMSD value of the re-docked tropolone is 1.038 Å.

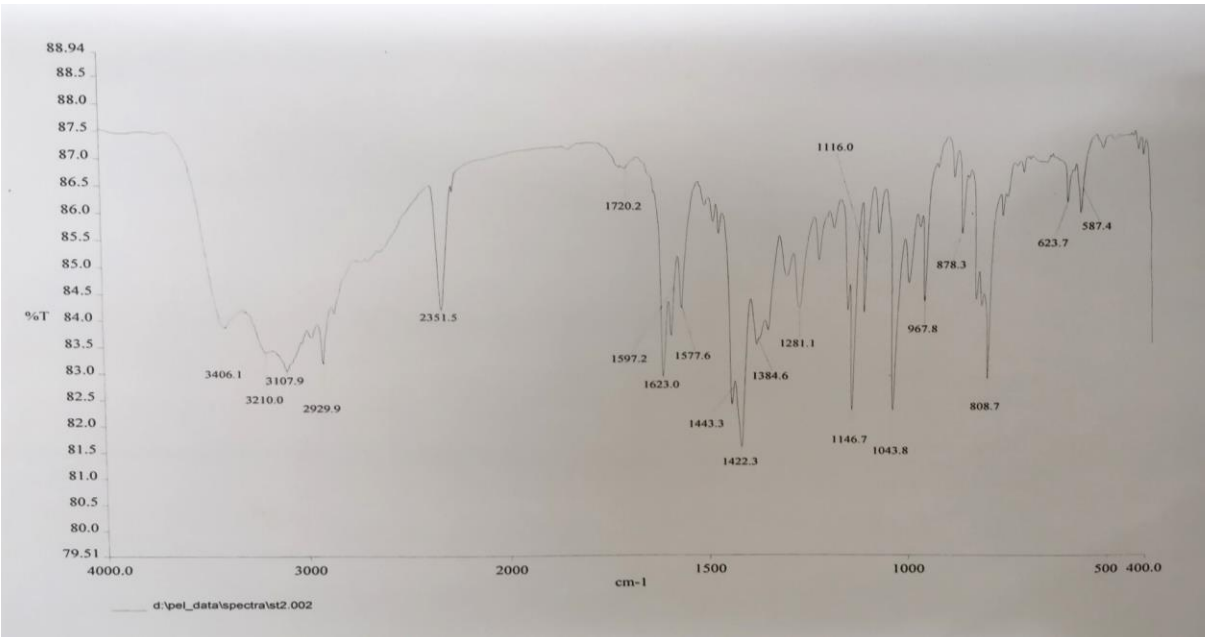

Figure S2. IR spectrum of ω-hydroxymoracin C (KBr disc)

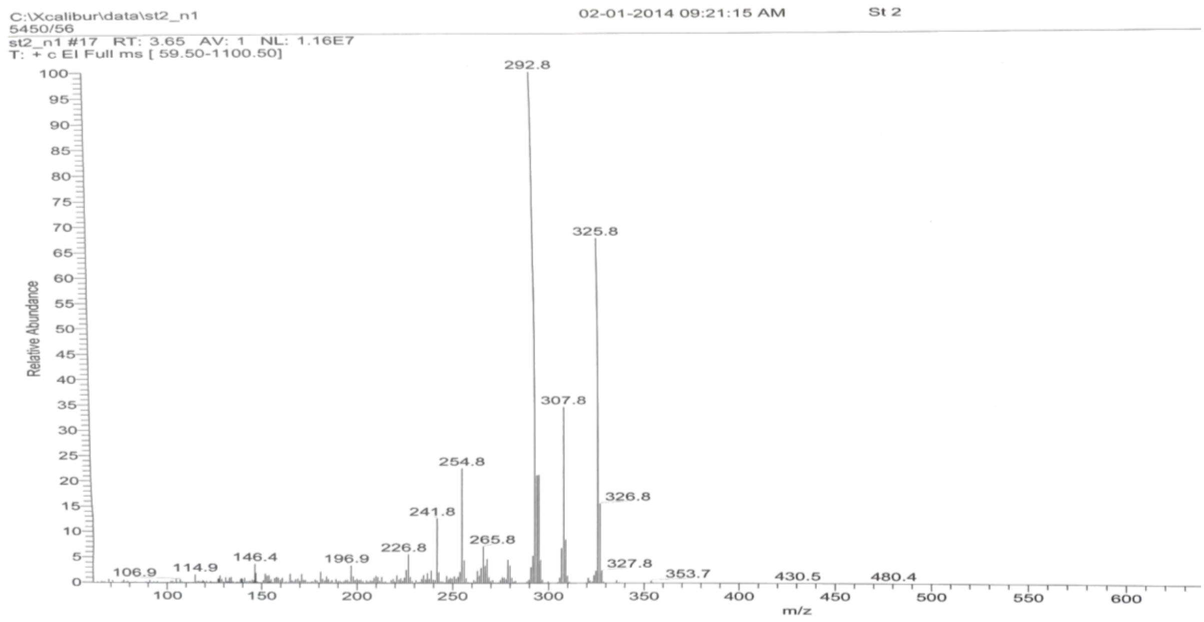

Figure S3. EI mass spectrum of ω-hydroxymoracin C

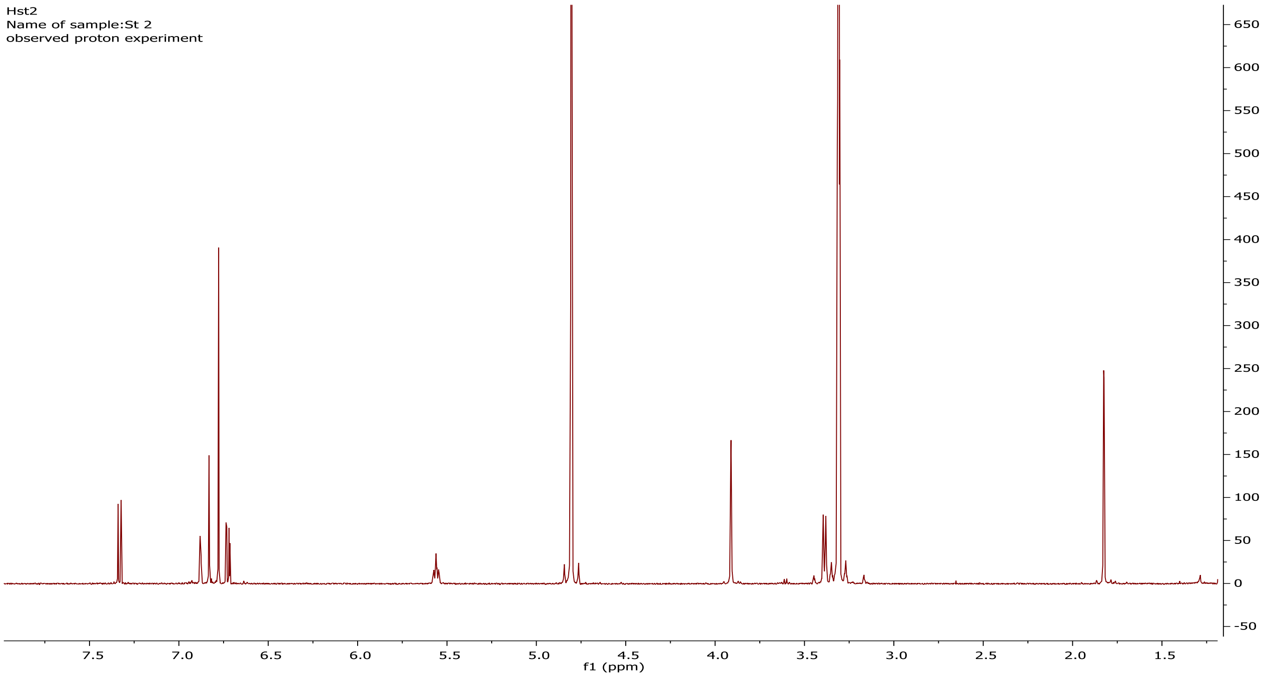

Figure S4. ^{1}H NMR spectrum of ω-hydroxymoracin C

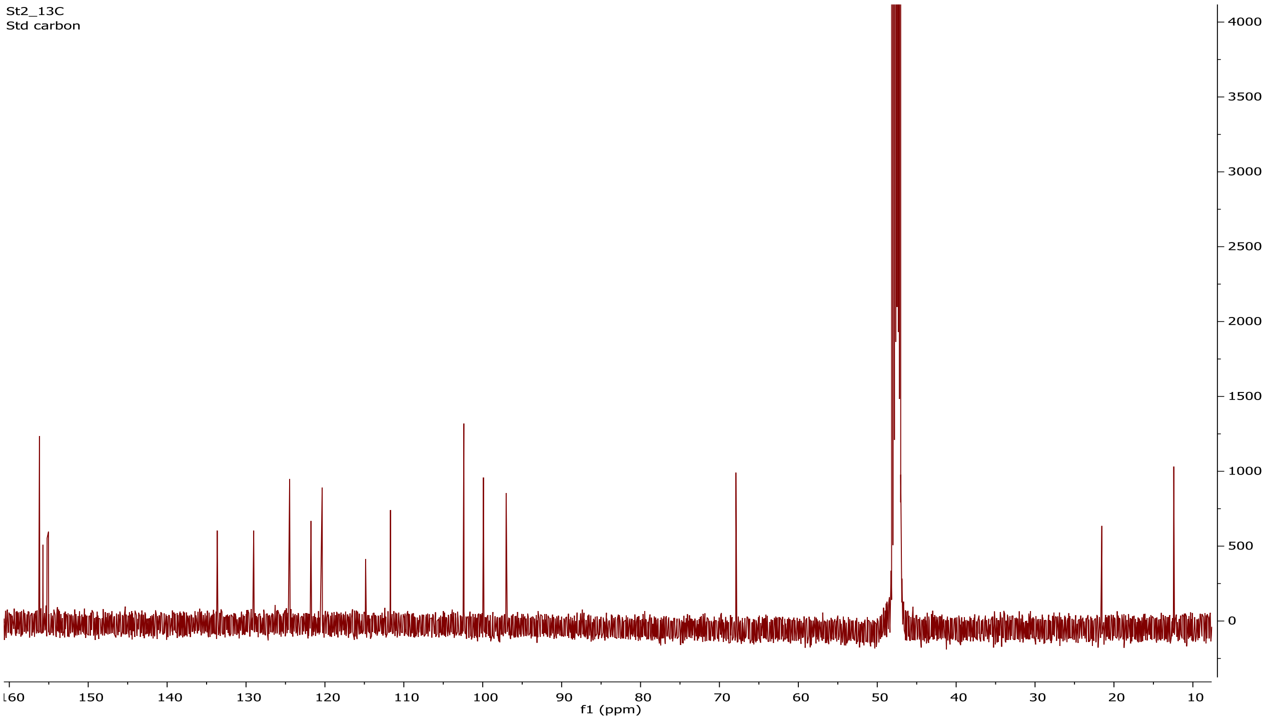

Figure S5. ^{13}C NMR spectrum of ω-hydroxymoracin C

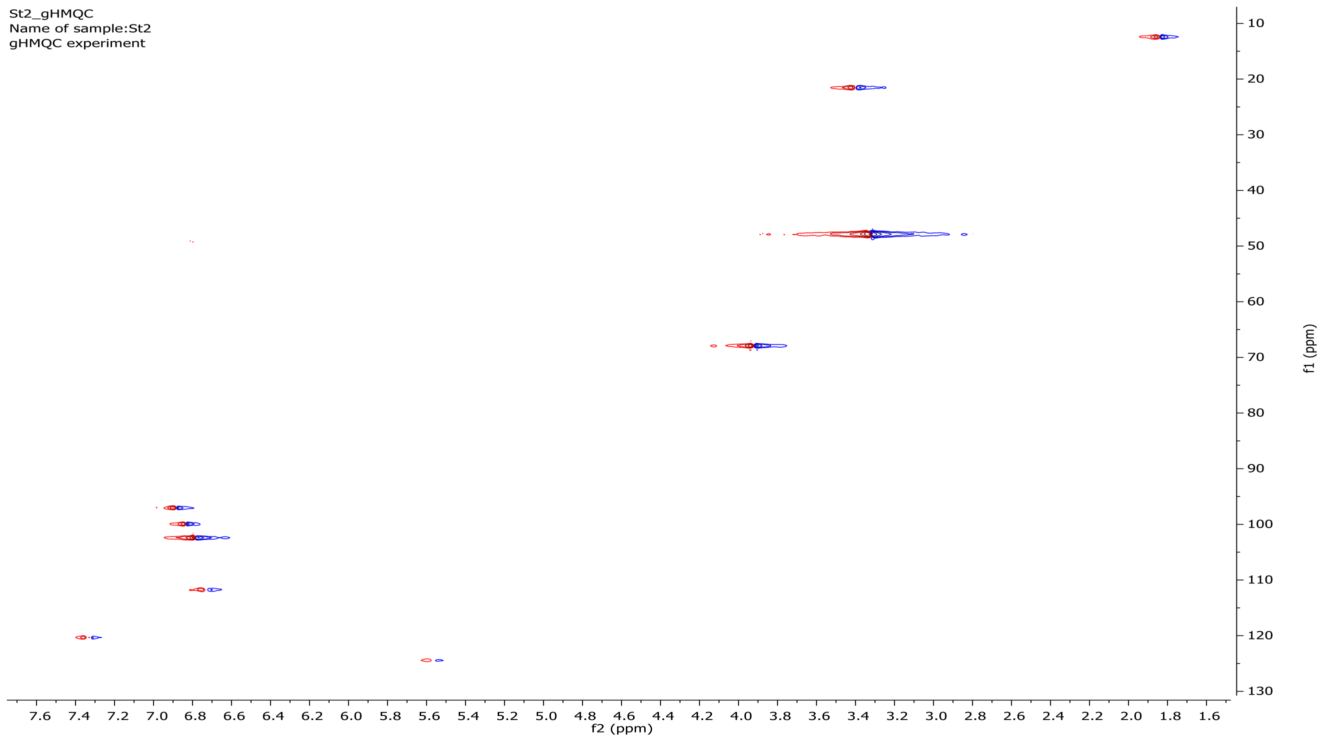

Figure S6. HMQC spectrum of ω-hydroxymoracin C

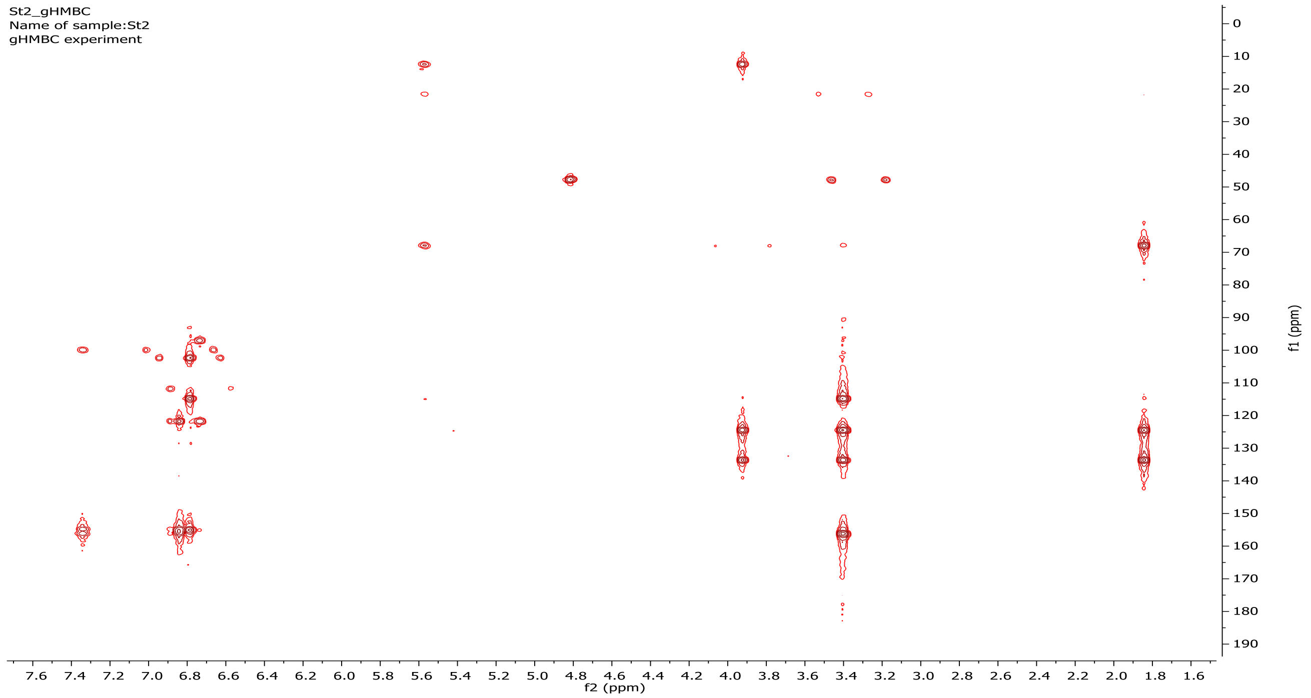

Figure S7. HMBC spectrum of ω-hydroxymoracin C

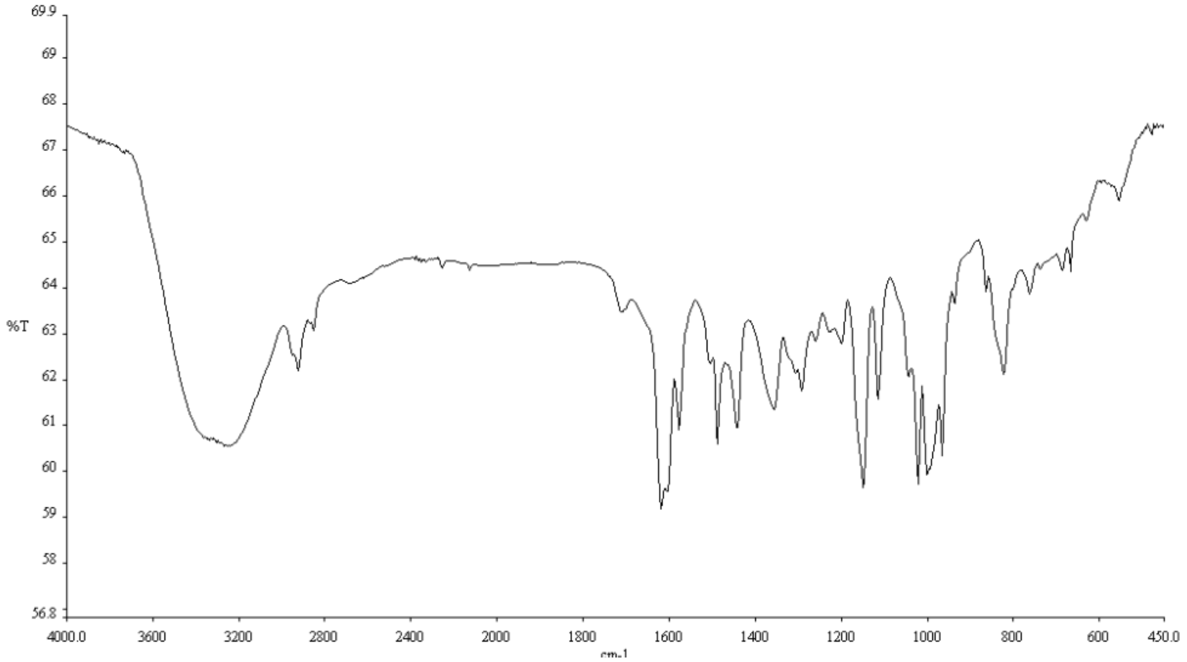

Figure S8. IR spectrum of moracin M (KBr disc)

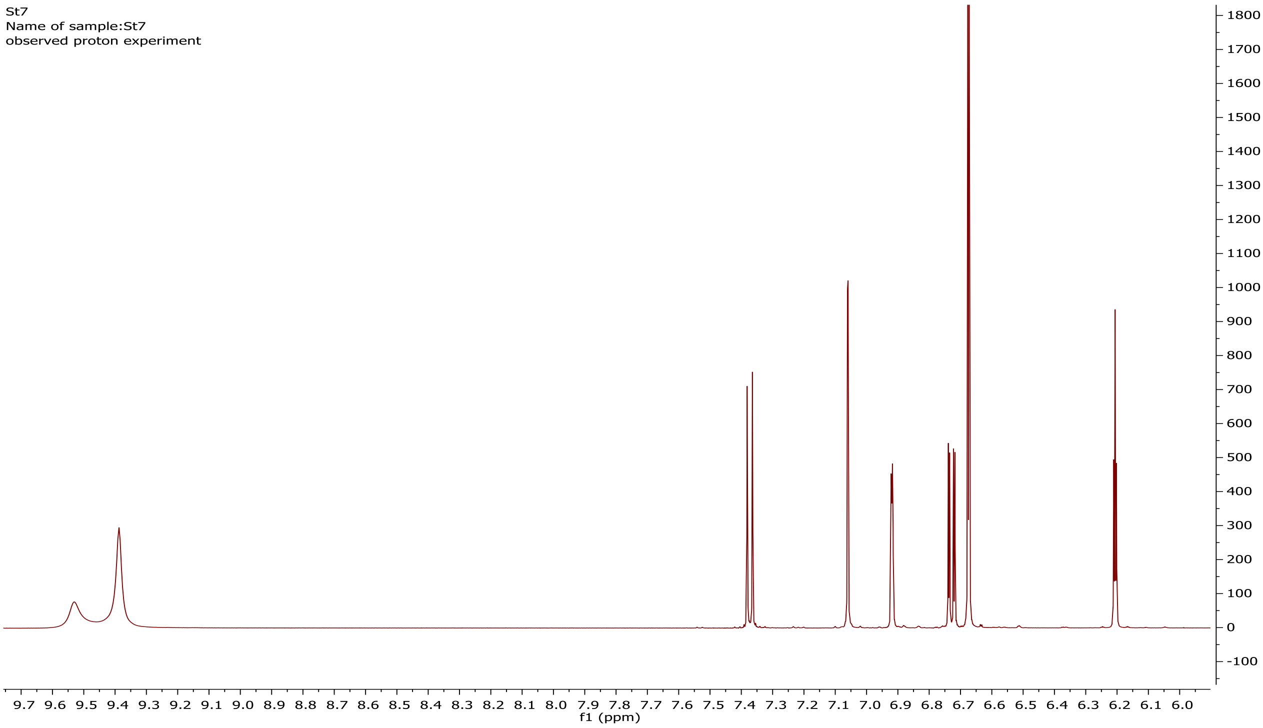

Figure S9. ^1H NMR spectrum of moracin M

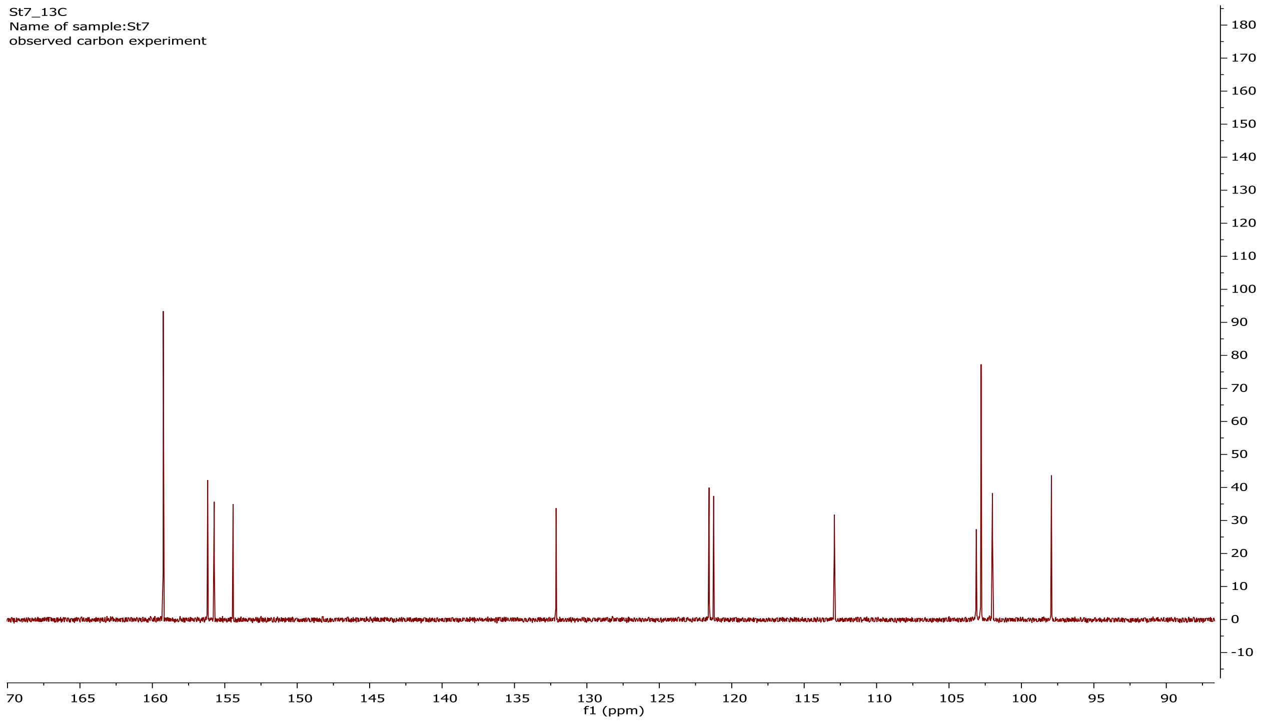

Figure S10. ^{13}C NMR spectrum of moracin M

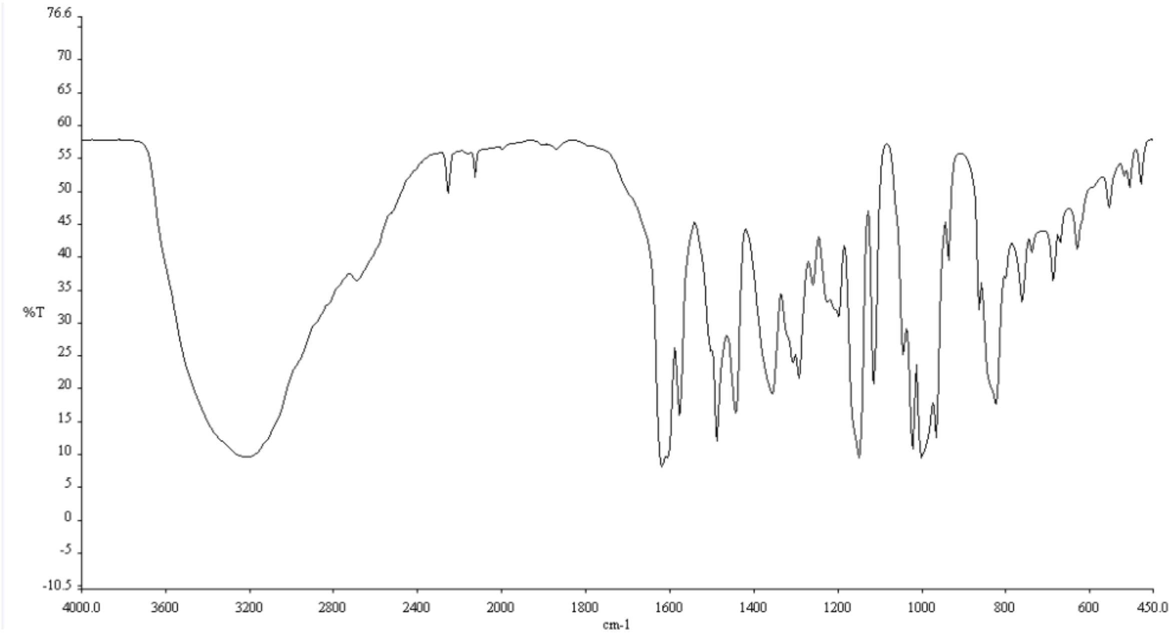

Figure S11. IR spectrum of moracin C (KBr disc)

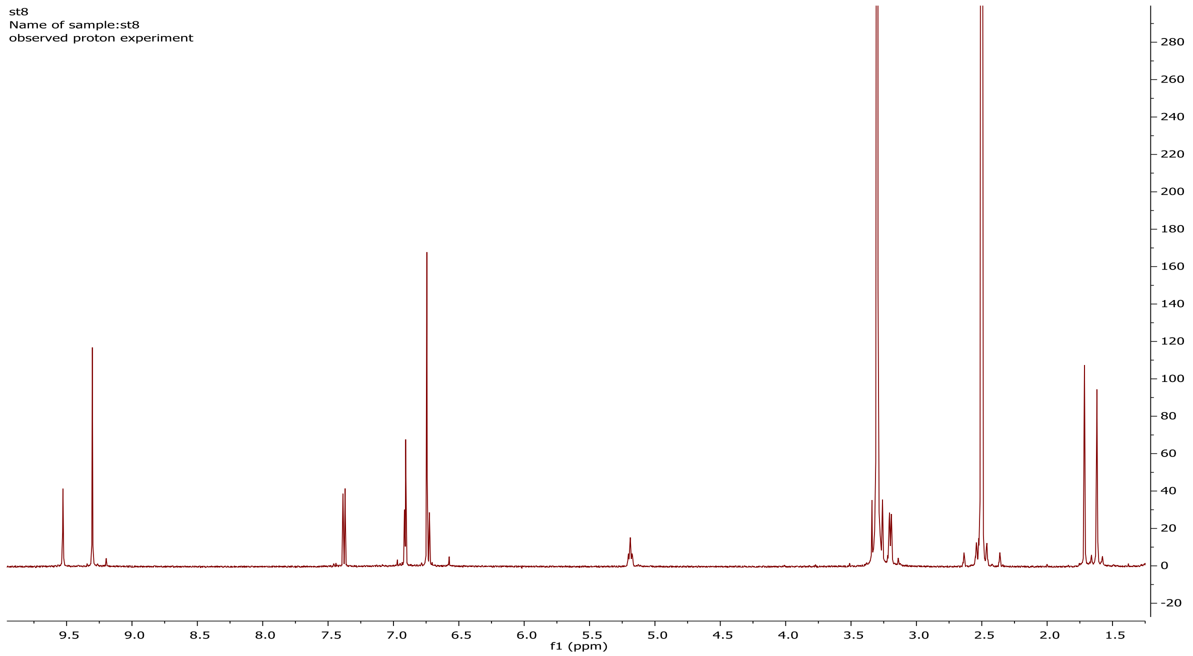

Figure S12. ^{1}H NMR spectrum of moracin C

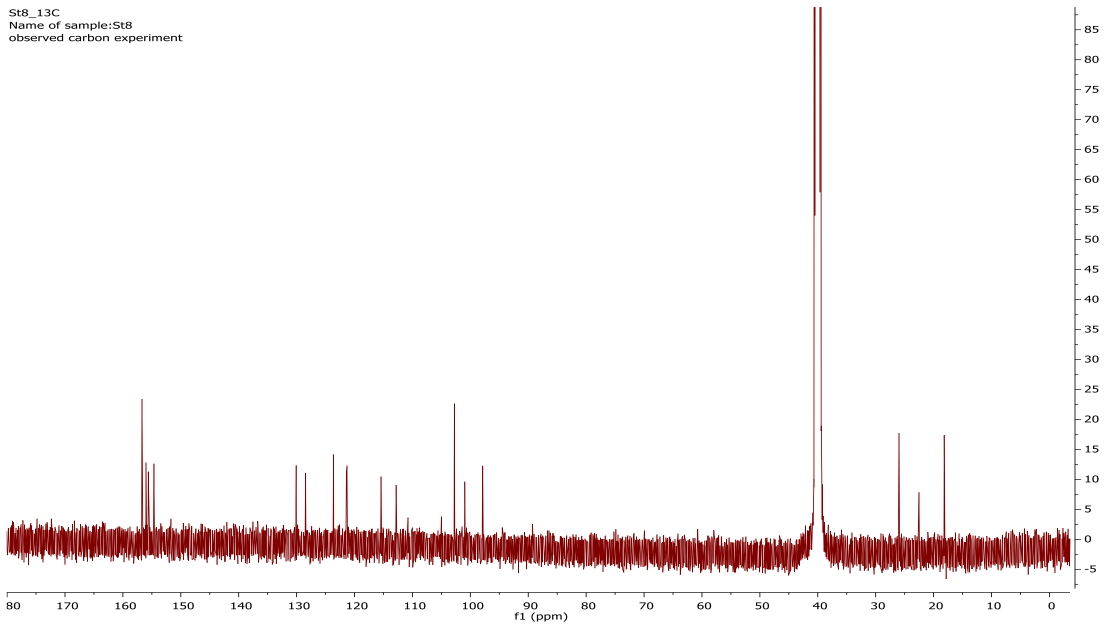

Figure S13. ^{13}C NMR spectrum of moracin C

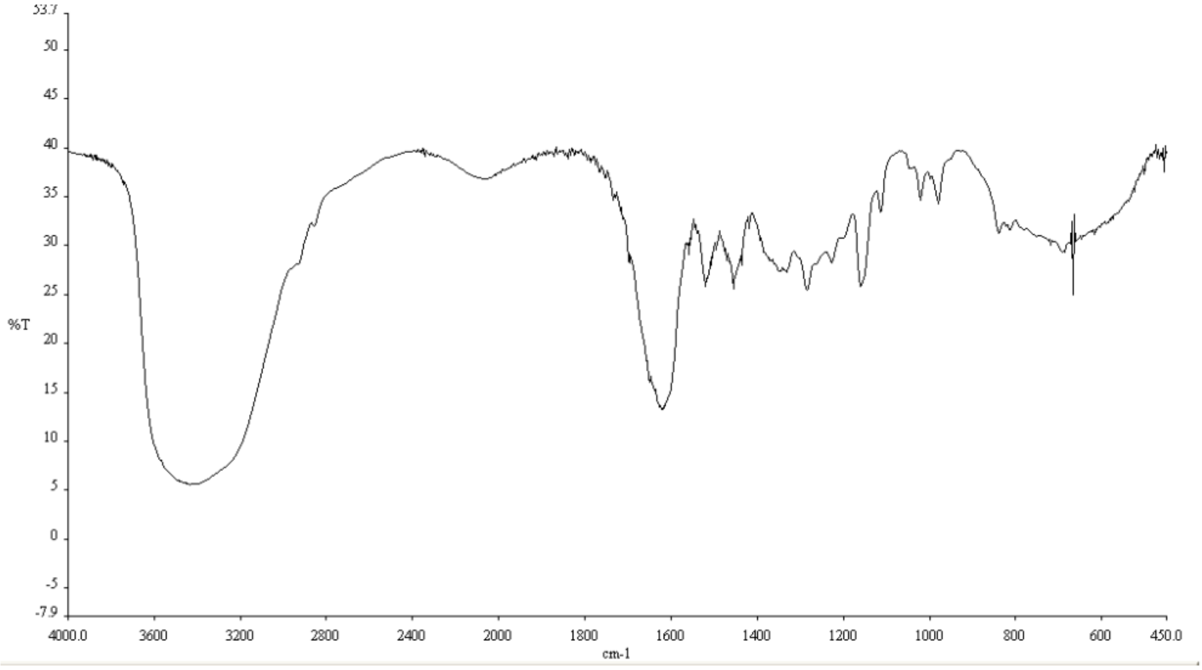

Figure S14. IR spectrum of 3, 4, 3′, 5′-tetrahydroxybibenzyl (KBr disc)

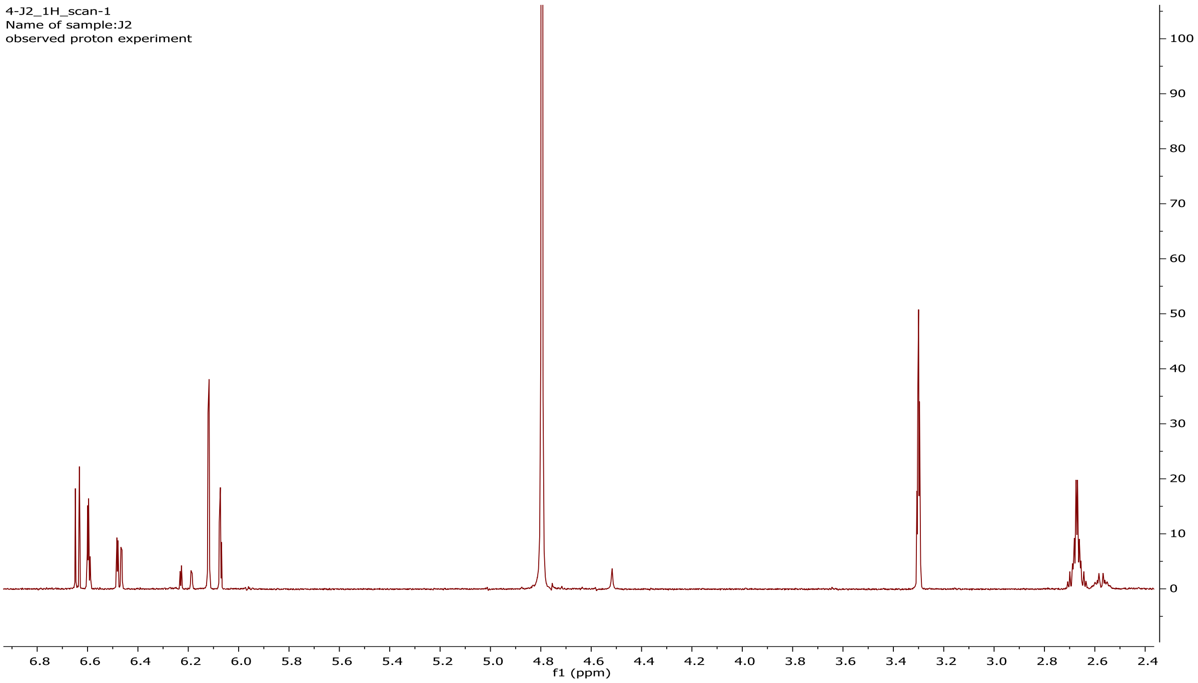

Figure S15. ^{1}H NMR spectrum of 3, 4, 3′, 5′-tetrahydroxybibenzyl

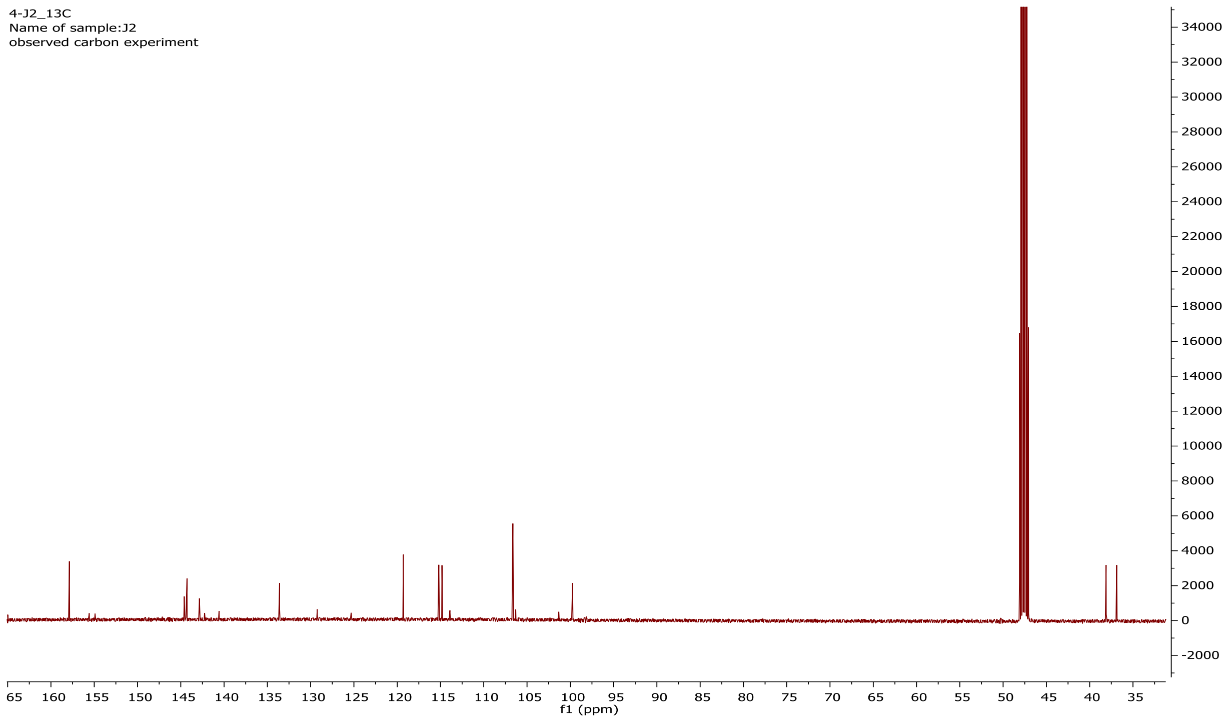

Figure S16. ^{13}C NMR spectrum of 3, 4, 3′, 5′-tetrahydroxybibenzyl

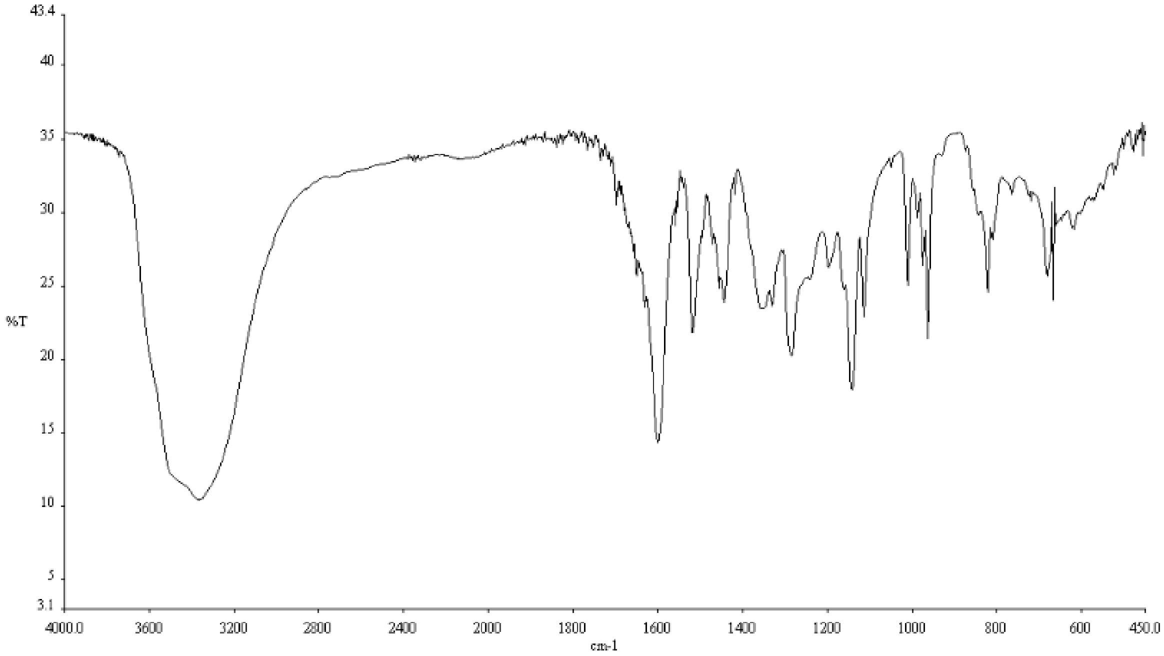

Figure S17. IR spectrum of piceatanol (KBr disc)

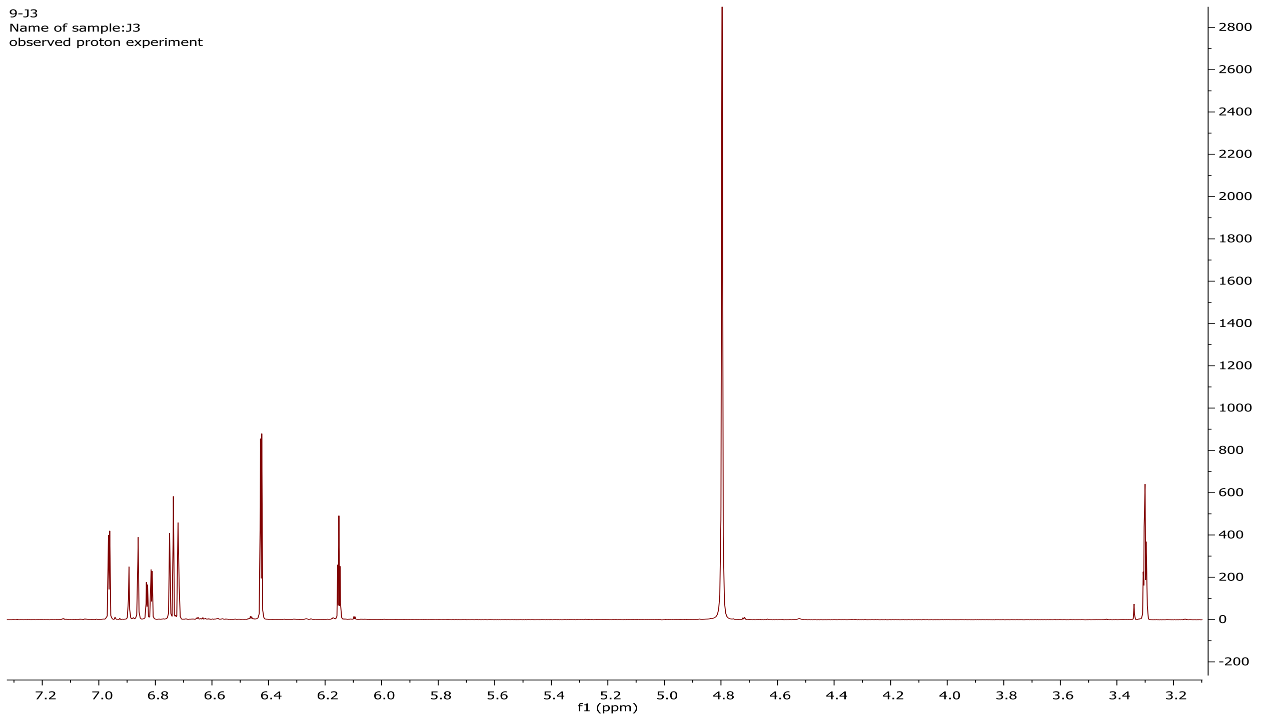

Figure S18. ^{1}H NMR spectrum of piceatanol
